# Inappropriate Antidiuretic Hormone Secretion and Cerebral Salt-Wasting Syndromes in Neurological Patients

**DOI:** 10.3389/fnins.2019.01170

**Published:** 2019-11-08

**Authors:** Haiying Cui, Guangyu He, Shuo Yang, You Lv, Zongmiao Jiang, Xiaokun Gang, Guixia Wang

**Affiliations:** Department of Endocrinology and Metabolism, The First Hospital of Jilin University, Changchun, China

**Keywords:** differential diagnosis, syndrome of inappropriate antidiuretic hormone secretion, cerebral salt-wasting syndrome, central nervous system, hyponatremia

## Abstract

The differential diagnosis of syndrome of inappropriate antidiuretic hormone secretion (SIADH) and cerebral salt-wasting syndrome (CSWS) in patients with neurological disorders has been a perplexing clinical controversy. The purpose of this review is to summarize the characteristics and risk factors of patients with different types of neurological disorders complicated by hyponatremia (HN) and review various methods to distinguish SIADH from CSWS. Common neurological disorders with high rates of HN include subarachnoid hemorrhage (SAH), traumatic brain injuries, stroke, cerebral tumors, central nervous system (CNS) infections, and Guillain-Barré syndrome (GBS), which have their own characteristics. Extracellular volume (ECV) status of patients is a key point to differentiate SIADH and CSWS, and a comprehensive assessment of relevant ECV indicators may be useful in differentiating these two syndromes. Besides, instead of monitoring the urinary sodium excretion, more attention should be paid to the total mass balance, including Na^+^, K^+^, Cl^−^, and extracellular fluid. Furthermore, the dynamic detection of fractional excretions (FE) of urate before and after correction of HN and a short-term infusion of isotonic saline solution may be useful in identifying the etiology of HN. As for brain natriuretic peptide (BNP) or N-terminal prohormone of BNP (NT-proBNP), more prospective studies and strong evidence are needed to determine whether there is a pertinent and clear difference between SIADH and CSWS.

## Introduction

Hyponatremia (HN), which is defined as serum sodium concentration <135 mmol/L, is the most common electrolyte disorder encountered in the clinical setting and is a frequent complication following neurological disorders. It has been reported that the HN accounts for 3–35% of inpatients (Sturdik et al., 2014; Holland-Bill et al., 2015; Gang et al., 2018), while the incidence in neurological patients has been reported to be as high as 50% (Rahman and Friedman, 2009). HN resulted in higher mortality, hospital costs, and readmission rates, and longer hospital stay (Deitelzweig et al., 2013; Hao et al., 2017; Althaus and Krapf, 2018). Syndrome of inappropriate antidiuretic hormone secretion (SIADH) and cerebral salt-wasting syndrome (CSWS) are the most common causes of HN in patients with neurological problems (Kalita et al., 2017; Moritz, 2019). In addition, SIADH and CSWS are two typical types of refractory HN, and the differential diagnosis between these two syndromes is difficult because of the overlapping signs, symptoms, and key laboratory data. Both syndromes show HN, low serum osmolality, and increased urinary sodium and urine osmolality (Peters et al., 1950; Schwartz et al., 1957). Besides the clinical manifestations of the primary disease, the symptoms of SIADH and CSWS are very similar, ranging from no symptoms with mild HN to serious impairment, including muscle cramps, seizures, coma, and even death, with severe HN (Maesaka et al., 2014; Monig and Arlt, 2017). The proper diagnosis of SIADH requires the detection of urine and serum osmolality, urinary sodium, cortisol, and thyroid hormone (Spasovski et al., 2014). However, a recent registry analysis (Greenberg et al., 2015), including 1,524 adjudicated adult patients with SIADH from 225 sites in the United States and the European Union showed that proper and comprehensive laboratory tests are only performed in 21% of identified SIADH patients. In addition, a recent study (Giuliani et al., 2013) supported by the Italian Endocrine Society found that less than half of clinicians surveyed were able to use validated biochemical parameters to diagnose SIADH. Besides, the treatment principles of SIADH and CSWS are contrary, which pose great challenges to clinicians. Therefore, the clinical differentiation of CSWS and SIADH is essential (Cuesta et al., 2016; Maesaka et al., 2018). The differentiation of these two syndromes has confused clinicians for more than 60 years as there is no consistent criteria, no available diagnostic tests in this field, and the effective and reliable method to distinguish CSWS from SIADH is limited (Peters et al., 1950; Schwartz et al., 1957; Cuesta et al., 2016). In this review, we will present the incidence and clinical characteristics of SIADH and CSWS in different types of neurological disorders, discuss the differences between these two types of refractory HN, and summarize the most pertinent differentiation diagnostic methods that may distinguish these two conditions of adult neurological disorders under present conditions.

## The Pathological Mechanism and Diagnostic Criteria of SIADH and CSWS

First described by Schwartz et al. (1957) in patients with lung bronchogenic carcinoma in 1957, SIADH was characterized by HN, high urine osmolality, and inappropriate concentrated urine in the absence of other causes of HN. HN secondary to SIADH is a result of water retention due to dysregulated release of antidiuretic hormone (ADH) (Zerbe et al., 1980) or gain-of-function mutations in the V_2_ vasopressin receptor in renal collecting tubules (Schwartz et al., 1957; Robertson, 2006). Below, we list the following features that were simplified as bedside criteria postulated by Misra et al. (2016), and at least two out of four of the following features were needed in a patient with HN to diagnose SIADH: (1) No sign of hypovolemia, such as hypotension, dry mucous membrane, tachycardia, or postural hypotension; (2) No laboratory evidence of dehydration, such as elevated hematocrit, hemoglobin, serum albumin, or blood urea; (3) Normal or positive fluid balance with absence of weight loss; (4) Central venous pressure (CVP) > 6 cm of water.

The concept of CSWS was proposed by Peters et al. (1950) in 1950 as an important cause of HN in patients with acute or chronic damage of the central nervous system (CNS), while it was abandoned for a long period of time. Nelson et al. (1981) questioned the diagnosis of 12 patients who fulfilled the laboratory criteria for SIADH but with significant decreases in their blood volume, which provided the clinicians a new understanding of CSWS. CSWS was characterized by negative sodium balance and volume depletion (Harrigan, 2001), although the precise pathophysiology of this condition is unclear, several studies have described the pathophysiology of the disease. First, the sympathetic nervous system hypothesis (Bitew et al., 2009) demonstrating that impaired sympathetic neural input to the juxtaglomerular apparatus may reduce proximal tubule sodium, urate, and water reabsorption and also decrease renin and aldosterone release. Second, natriuretic peptide theory (Berendes et al., 1997) indicated that the natriuretic factors, such as atrial natriuretic peptide (ANP) and brain natriuretic peptide (BNP), may play important roles in the occurrence and development of CSWS, leading to an increase in sodium excretion and urine volume.

Various diagnostic criteria were defined for CSWS (Singh et al., 2002; Yee et al., 2010; Leonard et al., 2015; Misra et al., 2016), however, which have not been completely unified. The early measurement of urine sodium and a completed documentation of increasing urinary output during the development of HN are extremely useful in the diagnosis of CSWS (Berkenbosch et al., 2002). Since CSWS was not well-defined and the diagnostic criteria are complicated, the authors present a simplified bedside criteria here (Misra et al., 2016), and the CSWS was considered in the presence of at least two out of four following features in a patient with HN: (1) clinical findings of hypovolemia, such as hypotension, dry mucous membranes, tachycardia, or postural hypotension; (2) laboratory evidence of dehydration, such as elevated hematocrit, hemoglobin, serum albumin, or blood urea nitrogen; (3) negative fluid balance as determined by intake output chart and/or weight loss; (4) CVP < 6 cm of water.

## The Incidence and Clinical Characteristics of SIADH and CSWS in Different Types of Neurological Disorders

Subarachnoid hemorrhage (SAH), traumatic brain injury (TBI), stroke, cerebral tumors, CNS infections, and Guillain-Barré syndrome (GBS) are classified as common neurological disorders with high rates of clinical HN (Karandanis and Shulman, 1976; Cuesta et al., 2016; Rumalla et al., 2017). The current review will provide an overview about the incidence and clinical characteristics of SIADH and CSWS in common types of neurological disorders (Table 1).

**Table 1:** The characteristics of different types of neurological disorders.

	**Incidence of HN**	**Risk factors**	**Occurred time**	**Etiology of HN**
SAH	40–57%	High-grade SAH; presence of anterior circulation aneurysms; in hydrocephalia; haptoglobin genotype associated with CSWS after SAH	Within the first 3 days to 1 week	SIADH was more common, but more severe Hunt and Hess grades were associated with CSWS
TBI	13.7–51%	Increased Rotterdam CT scores; the presence of cranial fractures; greater fluid intake from days 1 to 3	Within 3 days to the second week after injury	SIADH is the main cause; CSWS varies widely in TBI patients, from 0.8 to 34.6%
Stroke	12–43%	Stroke-associated HN is higher in acute geriatric medicine wards	Within the first week following sICH	The etiology of HN in patients following stroke is controversial; however, HN significantly affects the outcome of stroke especially when it is due to CSWS rather than SIADH
Cerebral tumor	8.4–35%	Old age (>60 years or ≤ 7 years), female sex, tumor size, rate of decline of blood sodium, low sodium concentration on postoperative days 1–2, and long operation time; patients with Cushing disease, craniopharyngioma or Rathke's cleft cyst	Delayed HN usually develops on postoperative days 4–10, and SIADH always occurred in days 7–14	SIADH was common in adult patients; however, CSWS was the main cause of HN among pediatric patients
CNS infections	38.7–73% in TBM, 4144.4% in TBE and may be as high as 59% in autoimmune encephalitis	The younger (<35 years) and elder (>60 years) TBE patients are more susceptible to HN; the use of IVIG may aggravate HN and the neurological symptoms	No further evidence was found	CSWS was the main cause of HN in adults with TBM, whereas SIADH was more common in children; dehydration seems to be the main cause of HN in TBE; the exact cause of HN in autoimmune encephalitis remains unclear
GBS	11.8–48%	Advanced age, deficiency anemia, alcohol abuse, hypertension, and the utilization of IVIG	Within an average of 8 days after GBS onset	SIADH is the main cause of HN in GBS; RSW has also been reported in GBS in a few cases

*HN, hyponatremia; SAH, subarachnoid hemorrhage; TBI, traumatic brain injury; sICH, spontaneous intracerebral hemorrhage; CNS, central nervous system; TBM, tuberculous meningitis; TBE, tick-borne encephalitis; IVIG, intravenous immunogloblin; GBS, Guillain-Barré syndrome; RSW, renal salt wasting; SIADH, syndrome of inappropriate antidiuretic hormone secretion; CSWS, cerebral salt-wasting syndrome*.

### Subarachnoid Hemorrhage

Previous studies have shown that 40–57% of patients who were admitted with SAH may develop HN, particularly in patients who were identified as having high-grade SAH, in the presence of anterior circulation aneurysms and in hydrocephalia (Sherlock et al., 2006; Hannon et al., 2014). It is estimated that 40% of patients may develop HN within the first 3 days of hemorrhage, while about 30% of patients develop HN 1 week after rupture occurred (Sherlock et al., 2006), which suggests that it is important to monitor the serum sodium changes within 1 week after SAH. The pathologic mechanisms underlying SAH-associated HN are not clear at present. There are different voices about the etiology of HN following SAH, some small patient cohort studies suggested that CSWS is the most common cause of HN following SAH (Palmer, 2000; Betjes, 2002). Ultimately, much more evidence demonstrated that most cases of HN after SAH are due to SIADH (Sherlock et al., 2006; Tisdall et al., 2006; Hannon et al., 2014). A previous study (Murthy et al., 2016) indicated that haptoglobin [an acute phase protein whose major function is to bind free hemoglobin (Rother et al., 2005) genotype] is an independent predictor of CSWS after SAH. Sherlock et al. (2006) indicated that the incidence of HN in patients was independent of whether who received interventional radiology, with coiling of aneurysms, or treated with craniotomy and clipping, and there was no clear pattern for those patients with HN ultimately ascribed to SIADH or CSWS. A retrospective analysis of 335 patients conducted by Hoffman et al. (2018) suggested that SIADH was more common than CSWS in anterior circulation aneurysms (90%) compared with posterior circulation aneurysms (75%). Besides, more severe Hunt and Hess grades were related to the development of CSWS rather than SIADH (*P* = 0.002) (Hoffman et al., 2018).

### Traumatic Brain Injury

HN is common in traumatic brain injuries (TBIs), occurring between 3 days and the second week after injury (Moro et al., 2007; Lohani and Devkota, 2011), with an incidence of 13.7–51% among high-risk patients (Moro et al., 2007; Yumoto et al., 2015). The following literatures demonstrated the definition of high risk. Lohani and Devkota (2011) reported that the increased Rotterdam CT scores are associated with a higher incidence of HN, and Yumoto et al. (2015) first reported that the presence of cranial fractures (*P* = 0.005) and greater fluid intake from days 1 to 3 (10,618 ml vs. 9,149 ml, *P* = 0.012) were found to be risk factors for HN in TBI patients. Moro et al. (2007) found that cerebral contusion and acute subdural hematoma are the two main causes of HN caused by TBI, which suggests that more attention is needed to monitor the serum sodium of patients with those two types of TBI. The two common causes of TBI-associated HN with natriuresis are SIADH and CSWS. Brain contusion and brain swelling caused by TBI may interfere and damage normal neuroendocrine function of the hypothalamus and pituitary system, therefore causing SIADH or CSWS and triggering central HN (Hannon et al., 2012; Taylor et al., 2017). Previous studies (Agha et al., 2005; Yumoto et al., 2015) have elaborated that SIADH is the main cause (over 80%) of HN of patients with TBIs. Leonard et al. (2015) found the incidence of CSWS varies widely in TBI patients, from 0.8 to 34.6%. Nevertheless, a recent report by Shen et al. (2017) presented the concurrent syndrome of SIADH and CSWS after TBIs in four patients, which poses to clinicians new ideas for the diagnosis and treatment of TBI-associated HN.

### Stroke

The world is facing an epidemic of stroke, which is the third most common cause of morbidity and mortality after coronary heart disease and cancer (Towfighi et al., 2010; Hankey, 2017). HN was reported to exist in 12–43% of stroke patients (Saleem et al., 2014; Kalita et al., 2017). The incidence of stroke-associated HN is higher in acute geriatric medicine wards (Hoyle et al., 2006). Gray et al. (2014) reported that majority of patients develop HN within the first week following spontaneous intracerebral hemorrhage (sICH). Unfortunately, we did not find additional information about ischemic stroke. The incidence of HN varies in different types of stroke, meaning sICH showed a higher ratio of incidence than that of ischemic stroke (Hannon and Thompson, 2014; Kalita et al., 2017). The etiology of HN in patients following stroke is controversial. Previous studies (Gray et al., 2014; Saleem et al., 2014) found that SIADH may be the predominant cause of stroke-associated HN, especially in sICH patients. However, a recent study showed an opposite result; Kalita et al. (2017) presented that CSWS is the most common cause of HN, and Saleem et al. (2014) reported that HN significantly affected the outcome of stroke especially when it is caused by CSWS rather than SIADH. Therefore, it is very important to monitor the trends in sodium levels closely, especially in elderly patients in the first week following sICH.

### Cerebral Tumors

Patients undergoing neurosurgery are always prone to develop HN, especially in patients with craniopharyngioma, Rathke's cleft cyst, and Cushing disease. Previous studies have shown that HN occurred in 8.4–35% cases (Hussain et al., 2013; Kiran et al., 2017). Previous studies demonstrated that old age (>60 years), gender, and tumor size were identified as potential predictors of delayed symptomatic HN (Cote et al., 2016; Yoon et al., 2019); meanwhile, researchers reported that the incidence of delayed HN in patients with non-functioning pituitary tumor who underwent endoscopic transsphenoidal surgery was 22.6%. Furthermore, they found that the rate of decline of blood sodium and low sodium concentration on postoperative days 1–2 and long operation time were associated with the development of delayed HN in such patients (Cote et al., 2016; Yoon et al., 2019). Delayed HN usually develops on postoperative days 4–10 (Jahangiri et al., 2013; Yoon et al., 2019). Kiran et al. (2017) reported that patients with HN caused by SIADH mostly occurred on days 7–14. CSWS was reported to be the most common cause of HN among pediatric patients following intracranial tumor surgery. Female children younger than 7 years old were considered important risk factors for CSWS (Williams et al., 2016). Unfortunately, available studies on etiology and clinical characteristics of HN in adult patients with cerebral tumor are quite limited. More studies are needed to confirm the theory.

### CNS Infections

Infectious meningitis, such as sepsis, bacterial meningitis, and tuberculous meningitis (TBM) and non-infectious conditions, such as autoimmune encephalitis, may result in a number of metabolic alterations, and HN is the most common electrolyte disorder in hospitalized patients (Karandanis and Shulman, 1976; von Vigier et al., 2001). Previous studies have shown that HN was more common in TBM than bacterial meningitis or aseptic meningitis, with an incidence of 38.7–73% among TBM patients (Karandanis and Shulman, 1976; Inamdar et al., 2016). In addition, CSWS was the most common cause of HN in TBM patients, and fludrocortisone was always used in refractory patients (Misra et al., 2016, 2019). Misra et al. (2016) demonstrated that CSWS was related to the severity of TBM, Glasgow Coma Scale (GCS) score, basal exudates, and infarctions. SIADH was reported in children with TBM. Cotton et al. (1991) indicated that SIADH occurs commonly in children with TBM, and its presence appears to influence the outcome of TBM in children. Moreover, the presence of SIADH was associated with a higher intracranial pressure, and fluid restriction is recommended (Cotton et al., 1993).

HN was reported to exist in 41–44.4% of tick-borne encephalitis (TBE) patients, and the younger (<35 years) and elder (>60 years) patients are more susceptible to HN. In addition, researchers concluded that dehydration seems to be the main cause of HN in the course of TBE (Czupryna et al., 2014, 2016), which suggests for us to pay more attention to estimate the blood volume and use hyperosmolar anti-edematous fluids, such as mannitol, cautiously. However, HN in encephalitis may also be caused by SIADH, with an incidence of 5% among TBE patients (Czupryna et al., 2014); their further research showed that urinary sodium excretion to plasma copeptin ratio may be used as a potential biomarker of SIADH in patients with TBE (Czupryna et al., 2017).

HN also can be observed in autoimmune encephalitis patients; however, study on the relationship between HN and autoimmune encephalitis is limited. One case series (Irani et al., 2010) showed that the prevalence of HN may be as high as 59% in autoimmune encephalitis patients; however, the exact cause of HN remains unclear. There are a few scattered reports identifying the association between anti-voltage-gated potassium channel limbic encephalitis and SIADH (McQuillan and Bargman, 2011; Lee et al., 2013; Black and Hamada, 2018). While McQuillan and Bargman (2011) indicated that the use of intravenous immunogloblin (IVIG) may aggravate HN and the neurological symptoms, which suggests to us that more attention is needed to the serum sodium level when we administering IVIG as a treatment regimen.

### Guillain-Barré Syndrome

Previous studies demonstrated that HN is common in GBS, with an incidence of 11.8–48% (Saifudheen et al., 2011; Hiew et al., 2016; Rumalla et al., 2017), but pseudohyponatremia caused by IVIG administration may confound the HN incidence (Colls, 2003; Steinberger et al., 2003). The evidence from the nationwide inpatient sample of 54,778 Guillain-Barré syndrome patients showed that HN was related to severe GBS and was independently associated with adverse discharge disposition (odds ratio: 2.07, 95% confidence interval: 1.91–2.25, *P* < 0.0001), which suggests that serum sodium should be carefully monitored in GBS patients, especially in patients with a high risk (advanced age, deficiency anemia, alcohol abuse, hypertension, and the utilization of IVIG, all *P* < 0.0001) (Rumalla et al., 2017). HN has been described mainly as SIADH (Inoue et al., 2010; Monzon Vazquez et al., 2011; Cakirgoz et al., 2014), and the degree of severity of SIADH was associated with the severity of GBS. In addition, it was reported that HN was detected at an average of 8 days after GBS onset (Saifudheen et al., 2011). However, there are still few cases of renal salt-wasting syndrome (RSW) in GBS patients (Cooper et al., 1965; Lenhard et al., 2011; Kumar et al., 2019). Kumar et al. (2019) reported that RSW can be diagnosed after a median of 4.5 (range 3–21) days of admission, and it may adversely affect the cardiovascular stability and outcome, which gave the clue that RSW may be an important complication in GBS patients and appeared to be closely associated with dysautonomia.

## Differentiating SIADH From CSWS in Patients With Neurological Disorders

The differentiation of SIADH from CSWS has been extremely difficult to accomplish due to the significant overlapping of clinical findings between both syndromes. As mentioned above, both syndromes are related to neurological disorders; normal renal, thyroid, and adrenal function; and hyponatremic and present inappropriate concentrated urines with high urinary sodium. In addition, both showed increased urine volume. Although the differentiation between the two syndromes is difficult, there are still some strategies worth studying. In the paper, we will review the most pertinent diagnostic methods that distinguish these two conditions of adult CNS disorders under present conditions.

### Determination of the State of Extracellular Volume in Differentiating CSWS From SIADH

The state of extracellular volume (ECV) is one of the factors that may help in distinguishing the two situations, being normal or increased volemia in SIADH and hypovolemic in CSWS. Radioisotope dilution techniques are the gold standard to assess blood volume, including 51 chromium-labeled red cells and iodine-131 or iodine-125 labeled to human serum albumin (HSA), and were first presented by Valdemar 100 years ago, reintroduced by Hart and Metz (1962). However, because of the high cost and complex testing procedures (Bartoli et al., 1993), it is currently used more in laboratory studies but not clinical trials. The blood volume of those two syndromes, therefore, is usually estimated by surrogates, as noted in Table 2 and should be evaluated comprehensively since these surrogates may be misleading as they are affected by many factors. The precise assessment of the ECV is the key to distinguish SIADH from CSWS. The symptoms of postural hypotension, increased heart rate, and dry skin mucosa may be helpful to diagnose CSWS. However, these symptoms can be affected by many factors (Table 2), and these differences are not always obvious, and there are errors in subjective judgments. In addition, absence of dehydration or hypovolemia in patients who are hospitalized at intensive care units and whose fluid statuses are closely monitored and fluid losses are replaced should not be excluded from the diagnosis of CSWS, and timely and early evaluation is particularly important for those patients. Therefore, further studies are needed to explore non-invasive, simple and convenient, and safe indicators or algorithms to assess blood volume.

**Table 2:** Comparison of the clinical and laboratory indicators of CSWS and SIADH.

	**SIADH**	**CSWS**	**Affect factors and limitations of clinical utility**
Related to blood volume			
Blood pressure	Normal/increased	Decreased/orthopedic hypotension	Stress state; measurement error
Heart rate	Normal	Increased	Nutrition state, history of liver disease
Body weight	Normal/increased	Decreased	Bed rest >1 week; caloric intake (Greenleaf et al., 1977)
BUN	Normal/decreased	Increased	Caloric intake
Albumin	Normal	Increased	Nutrition state, history of liver disease
HCT	Normal	Increased	Anemia; history of choric heart and lung disease
Wedge pressure	Normal/slightly	Decreased	History of heart disease; invasive method
CVP	Normal/slightly increased	Decreased	Intrathoracic pressure, inotropic efficacy, and compliance of the venous system (Shippy et al., 1984); invasive method
Serum UA concentration	Decrease	Decrease	Purines intake; renal function; history of hyperuricemia
Skin and mucosa	Normal	Dry	Subjective factor; age-related changes; status of nutrition
Related to sodium balance			
Urine volume	Normal/decrease	High	The administration of diuretics or antihypertensives; age; change in volume status
Urine sodium concentration	>30 mmol/l	>>30 mmol/l	Supplement of sodium; the administration of diuretics
BNP	Normal	Increased	History of heart and lung disease; age related; stress state

*CVP, central venous pressure; HCT, hematocrit; BUN, blood urea nitrogen; UA, uric acid; BNP, brain natriuretic peptide; SIADH, syndrome of inappropriate antidiuretic hormone secretion; CSWS, cerebral salt-wasting syndrome*.

### Is There a True Deficit of Na^+^ in the Circulation?

The common feature of both SIADH and CSWS is the presence of HN; however, the causes of HN in these two syndromes are different. Based on the pathogenesis of these two syndromes, patients with CSWS showed excessive water and sodium excretion by the kidney, whereas patients with SIADH showed increased water retention by the kidney. Berkenbosch et al. (2002) have identified the role that early quantitation of urine volume and urine sodium concentration may rapidly establish the correct diagnosis. Arieff et al. (2017) also indicated that in patients with HN complicated with a cerebral lesion, the detection of urine volume and sodium excretion is critical. Therefore, intake-output chart may help in differentiating CSWS from SIADH; in addition, polyuria and negative fluid balance from a chart is a feasible bedside measure. Patients with CSWS manifested markedly elevated urine volume and sodium excretion when compared to the counterpart. The elevated urine Na excretion may reflect the balance of sodium in the body to some extent; nevertheless, to determine whether there is a true deficit of Na^+^, mass balances rather than excretion rates of Na^+^ should be detected (Carlotti et al., 2001), and mass balance for Na^+^ + K^+^ rather than just for Na^+^ should be included since Na^+^ may enter into cells in conjunction with K^+^ exit. CSWS is a common exclusion of diagnosis; overall balances including Na^+^, K^+^, Cl^−^, and extracellular fluid must be evaluated, and the diagnosis should not be made in a patient without a true deficit of Na^+^ + K^+^.

### Value of Determining Fractional Excretion of Urate in Differentiating SIADH From CSWS

Previous studies found that serum uric acid levels are generally decreased and fractional excretion (FE) urate is increased in both SIADH and CSWS; however, hypouricemia increased and still showed in CSWS but not in SIADH after the correction of HN (Schwartz et al., 1957; Maesaka et al., 2009). Researchers found that V1 receptor stimulation may play a central role in inducing hypouricemia in SIADH; however, the mechanism of increased FEurate in CSWS remains unclear (Taniguchi et al., 2016).

FEurate can be calculated with simultaneous blood and urine collection by the following standard formula (Maesaka et al., 2014): FEurate = Uurate/Surate ÷ UCr/SCr × 100 (U = urine, S = serum, and Cr = creatinine). Normal value of FEurate is about 4–11%. It is suggested that FEurate can be used in the algorithm to differentiate SIADH from CSWS (Sonnenblick and Rosin, 1988; Maesaka et al., 2014). In most cases, the level of FEurate is more than 11%, and in SIADH, correction of HN will normalize FEurate to 4–11%; in contrast, it will continue to rise to higher than 11% in CSWS (Table 3). Consequently, the dynamic detection of FEurate may be a feasible method to identify the etiology for patients with intractable HN.

**Table 3:** Changes of FEurate in SIADH and CSWS after correction of HN.

**FEurate (normal value 4–11%)**	**CSWS**	**SIADH**
Before correction of HN	>11%	>11%
After correction of HN	>11%	4–11%

*SIADH, syndrome of inappropriate antidiuretic hormone secretion; CSWS, cerebral salt-wasting syndrome*.

### Can BNP Be Used as an Indicator in Diagnosing?

A previous study (Berendes et al., 1997) has shown that BNP may be a contributing factor of CSWS. Recently, a 30-month cohort study conducted by Tobin et al. (2018) presented that N-terminal prohormone of brain natriuretic peptide (NT-proBNP) is a quick and convenient assay to differentiate CSWS from SIADH; the cutoff value is 125 pg/ml, and the positive predictive value of NT-proBNP to detect hypovolemia in patients with CSWS was 93.33%, while the negative predictive value was 87.50%. In addition, Bunnag and Pattanasombatsakul (2012) reported that plasma NT-proBNP levels provide objective information with respect to volume status, and the best cutoff value of plasma NT-proBNP level to distinguish hypovolemic from euvolemic HN was 518 pg/ml, with a sensitivity 94.4% and a specificity 100%. However, a study by Misra et al. (2018a) showed that ANP and BNP levels were increased during HN and remained high even after correction of HN in tuberculous meningitis and acute encephalitis syndrome, especially in patients with CSWS, but there was no significant difference between CSWS and SIADH groups. NT-proBNP is a high variable indicator with short half-life and can be affected by many common settings, such as in patients with pulmonary diseases or extremely young or old age (Raymond et al., 2003; Das et al., 2005); they concluded that ANP and BNP levels could not be used to differentiate CSWS from SIADH. Finally, it remains controversial whether BNP can serve as an enabling factor for the differential diagnosis of the two syndromes. Prospective studies with a larger sample size are still needed for dynamic monitoring of BNP levels throughout the course of the disease.

### The Feasibility of Diagnostic Treatment

Fluid restriction is recommended for treating HN in uncomplicated SIADH (Schwartz et al., 1957; Spasovski et al., 2014); however, it may aggravate the cerebral lesion in a patient with complicated HN caused by neurological diseases. In the absence of a well-established cause of HN, fluid restriction may lead to cerebrovascular depletion and cerebral vasospasm (Upadhyay and Gormley, 2012), indicating that fluid restriction is not a safe and feasible method to differentiate intractable HN. In addition, supplement with isotonic saline is not always effective in improving serum sodium levels in SIADH, while it can restore the decreased serum sodium levels and intravascular volume in most CSWS patients (Harrigan, 2001; Yee et al., 2010). Therefore, a short-term infusion of isotonic saline solution, which is expected to improve the serum sodium in CSWS but not in SIADH, may serve as a method of identification. However, single isotonic fluids are often insufficient for the treatment of CSWS; usually, hypertonic saline has to be used, and sometimes fludrocortisone should be used in some refractory cases (Misra et al., 2018b).

## Treatment

The treatment of CSWS consists of volume replacement and the correction of HN with 0.9% sodium chloride and/or hypertonic saline (Spasovski et al., 2014). However, in some cases, this may not be able to correct the refractory HN. Fludrocortisone, as a type of mineralocorticoid hormone, has been reported to effectively control natriuresis (Sakarcan and Bocchini, 1998). This hormone was first reported for the treatment of CSWS in the 1980s in three elderly patients with head injury. As a result, all three patients responded well to fludrocortisone therapy (Ishikawa et al., 1987). Single reports of its administration in CSWS patients have appeared sporadically (Berendes et al., 1992; Kinik et al., 2001; Lee et al., 2008; Gurnurkar et al., 2018). Following the treatment of fluid and salt replacement, mineralocorticoid supplement also seems to be a safe and effective treatment for CSWS. Misra et al. (2018b) conducted a recent randomized clinical trial and provided class II evidence on the role of fludrocortisone in the treatment of HN related to CSWS in TBM patients. The results suggested that fludrocortisone (at an oral dose of 0.1–0.4 mg per day and starts with a small dose) may lead to earlier normalization of serum sodium levels, without affecting outcomes at 6 months, and the results are consistent with those of a randomized clinical trial in SAH patients with CSWS (Hasan et al., 1989).

Fluid restriction is recommended as first-line treatment in chronic moderate or profound HN caused by SIADH (Spasovski et al., 2014); however, fluid restriction may be insufficient or impractical. Tolvaptan, an oral vasopressin V2-receptor antagonist, might be an attractive option for correcting the HN due to SIADH. The SALT-1 and SALT-2 trials have shown that serum sodium can be safely improved at day 4 and 30 by the administration of tolvaptan in patients with SIADH (Schrier et al., 2006). The role of tolvaptan in the treatment of SIADH is indisputable; however, the cost of therapy and the need for long-term safety data may limit its widespread use. Therefore, several precautions are needed for using tolvaptan: (1) Tolvaptan should be initiated at a small dose and should not be used in conjunction with fluid restriction. In addition, it is important to monitor the serum sodium closely (recommended every 4–6 h after starting treatment) in case of an overly rapid correction (Cuesta and Thompson, 2016; Thajudeen and Salahudeen, 2016); (2) It is recommended to test liver function before the administration of tolvaptan due to its potential liver toxicity with chronic use (Peri and Giuliani, 2014). Tolvaptan may serve as a double-sided sword because of its high risk of rapid correction of HN and the potential liver toxicity. However, giving a bolus of saline is beneficial before restriction of fluid or challenging with tolvaptan (Cuesta and Thompson, 2016).

## Summary and Conclusions

**Figure 1:**
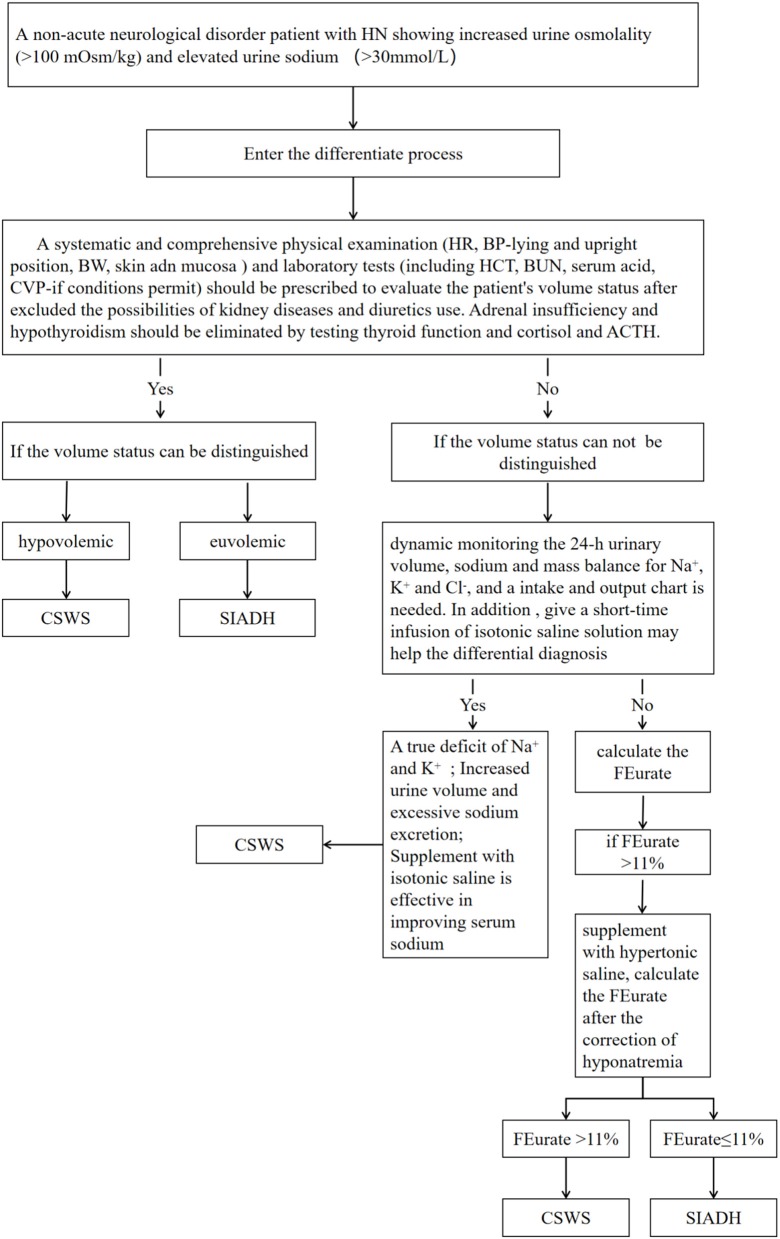
The process to differentiate cerebral salt-wasting syndrome (CSWS) from syndrome of inappropriate antidiuretic hormone secretion (SIADH).

No single and simple cause is involved in the pathophysiology of CSWS. With respect to the classical manifestations considered in the differential diagnosis of CSWS and SIADH, not all patients with increased BNP levels should be diagnosed with CSWS. CSWS should not be excluded in patients only with decreased uric acid levels as well. The differential diagnosis should be considered comprehensively, and after the literature review, we designed a process to differentiate SIADH and CSWS in Figure 1. To diagnose a non-acute neurological disorder patient with HN showing increased urine osmolality and elevated urine sodium concentration, a systematic and comprehensive physical examination and laboratory tests should be prescribed to evaluate the patient's volume status after excluding the possibilities of kidney diseases and diuretics use. Adrenal insufficiency and hypothyroidism should be eliminated when diagnosing. In addition, the dynamic detection of 24-h urinary volume and mass balance of Na^+^, K^+^, and Cl^−^ is an important indicator to distinguish both syndromes; meanwhile, short-term infusion of isotonic saline solution may be helpful to differentiate SIADH and CSWS. Besides, dynamically calculated FEurate before and after the correction of HN may also contribute to distinguish the two syndromes. Using the above method properly may lead to a valuable differential diagnosis.

In conclusion, the ECV status of patients is a key factor to differentiate SIADH and CSWS, despite it being difficult to evaluate in clinical practices. Instead of monitoring the urinary Na excretion, more attention should be paid to the total mass balance, including Na^+^, K^+^, Cl^−^ and extracellular fluid. Furthermore, the dynamic detection of FEurate before and after correction of HN and a short-term infusion of isotonic saline solution may be useful in identifying the etiology of HN. As for BNP or NT-proBNP, further prospective studies and strong evidence are needed to determine whether there is a pertinent and clear difference between SIADH and CSWS.

## Author Contributions

GW devised the main concept of the manuscript. HC and XG collected data and wrote the first draft of the manuscript. GH and YL contributed to editing this work. SY and ZJ contributed to the revision of draft. All the authors have read and approved the submitted version.

### Conflict of Interest

The authors declare that the research was conducted in the absence of any commercial or financial relationships that could be construed as a potential conflict of interest.
